# Supplementary material for: Reaction Mechanisms of H2S Oxidation by Naphthoquinones
Source: Antioxidants (Basel). 2024 May 20;13(5):619. doi: 10.3390/antiox13050619 (PMC11117753; doi:10.3390/antiox13050619)
Supplement: Supplementary file 1 [file antioxidants-13-00619-s001.zip › antioxidants-2982542-supplementary.pdf]

antioxidants

Supplemental Information

Reaction Mechanisms of H_2S Oxidation by Naphthoquinones

Kenneth R. Olson [a,b,*] Kasey J. Clear [c], Tsuyoshi Takata [a,b], Yan Gao [a], Zhilin Ma [a,b], Ella Pfaff [a,b], Anthony Travlos [a,b], Jennifer Luu [a,b], Katherine Wilson [a,b], Zachary Joseph [a,b], Ian Kyle [a,b], Stephen M. Kasko [a,b], Prentiss Jones Jr [d], Jon Fukuto [e], Ming Xian [f], Gang Wu [g] and Karl D. Straub [h,i]

a. Indiana University School of Medicine - South Bend Center, South Bend, Indiana 46617 USA
b. Department of Biological Sciences, University of Notre Dame, Notre Dame, IN 46556, USA
c. Department of Chemistry and Biochemistry, Indiana University South Bend, South Bend, Indiana 46615 USA
d. Toxicology Department, Western Michigan University Homer Stryker M.D. School of Medicine, Kalamazoo, MI 49007 USA
e. Department of Chemistry, Sonoma State University, Rohnert Park, CA 94928, USA
f. Department of Chemistry, Brown University, Providence, RI 02912, USA
g. Department of Internal Medicine, University of Texas-McGovern Medical School, Houston, Texas 77030, USA
h. Central Arkansas Veteran's Healthcare System, Little Rock, AR 72205, USA
i. Departments of Medicine and Biochemistry, University of Arkansas for Medical Sciences, Little Rock, AR 72202, USA
* Correspondence: **correspondence to:** olson.1@nd.edu; **Phone:** (574) 631-7560; **Fax:** (574) 631-7821

Supplemental Figures

Figure S1. Typical experiment showing the change in AzMC fluorescence as a function of time after the addition of H_2S and various naphthoquinones (NQs). Traces show AzMC fluorescence for 300 μM H_2S alone and after the addition of 3 μM 1,4-naphthoquinone (1,4-NQ), 3 μM juglone (Jug), 3 μM plumbagin (PB), 10 μM menadione (Mena), 10 μM 2-methoxy-1,4-naphthoquinone (2-MNQ), or 3 μM 2,3-dimethoxy-1,4-naphthoquinone (DMNQ). H_2S and naphthoquinones were added to five 96-well plates at t = 0 and the plates were covered with tape. The tape was removed and AzMC (25 μM) was added at t = 15, 30, 60, 120, and 240 min; then, the plates were re-taped, and fluorescence was measured 10 min after adding AzMC. Values are expressed as mean +SE, n = 4 wells per experiment. In most instances, SE is given by a symbol.

Figure S2. Effects of tempol on the consumption of 300 μM H_2S by 3 μM 1,4-NQ (**A**), 3 μM DMNQ (**B**), or 1 μM DCNQ (**C**). Tempol alone concentration-dependently inhibited H_2S consumption, preventing further analysis of its effects on H_2S consumption by NQs.

Figure S3. Traces of oxygen consumption following serial additions of 100 μM H_2S (down arrows) to 100 μM 1,4-NQ (**A**), juglone (**B**), plumbagin (**C**), DMNQ (**D**), or DCNQ (**E**). (**F**) Summary of oxygen consumed by each addition of H_2S. (**G**) Summary of the rate of oxygen consumption during the initial 2 min after H_2S addition. (**H**) Traces of oxygen consumed with the addition of 50-300 μM H_2S to the buffer (italic values in brackets) or to 100 μM DCNQ. (**I**) Diffusion of oxygen into the chamber after the addition of 400 μM H_2S to 100 μM DCNQ (DCNQ+H_2S) or buffer sparged with nitrogen prior to placement in the chamber (N_2 out) or while in the chamber (N_2). The rates of oxygen diffusion were similar, at 0.94, 0.93, and 0.93 μmol·min^{-1}, respectively. (**F, G**) Mean +SE *(n=3)*, where error bars may be within the symbol. (**J-O**) Oxygen consumption by 10 μM NQs and 300 μM H_2S does not exhibit a lag phase.

Figure S4. Effects of thiol adducts on the NQ-catalyzed consumption of H_2S. Here, 1 mM NQ was incubated with 1 mM of either GSH or cysteine (Cys) for 1 h, then diluted to a lower NQ concentration (indicated above each figure). These concentrations were chosen to provide ample time to see a potential increase or decrease in H_2S consumption (compare 3 μM to 1 μM 1,4-NQ in top left panels). GSH and cysteine controls were diluted to the same final concentration as the NQs. GSH had minimal effect on H_2S consumption by 1,4-NQ or DCNQ, slightly decreased consumption by juglone and DMNQ, and increased consumption by plumbagin, menadione, and 2-MNQ. Cysteine decreased H_2S consumption by 1,4-NQ, juglone, and DCNQ, slightly increased it by menadione,

and had no effect with plumbagin, 2-MNQ, or DMNQ. Values expressed as the percent of H_2S removed from the solution by NQs, calculated from an average of four replicates.

Figure S5. Effects of propylamine (PA) NQ adducts on H_2S or oxygen consumption. (**A, B**) Here, 5 mM 1,4-NQ or 1 mM 1,4-NQ was incubated without or with 5 mM PA (1:1 or 1:5 NQ:PA in **A** and **B**, respectively) overnight, then diluted to either 3 μM, 10 μM, or 30 μM NQ, and consumption of 300 μM H_2S was measured with AzMC over 240 min. The effect of PA on H_2S consumption was inversely related to the 1,4-NQ concentration when the NQ:PA incubation ratio was 1:1 but essentially unaffected at a 1:5 NP:PA ratio. The effects of juglone (Jug, **C**), menadione (Mena, **D**), plumbagin (Plum, **E**), and 2,3-dimethoxy-1,4-NQ (DMNQ, **F**) on H_2S consumption were as follows: NQs (5 mM) were incubated with 5 mM PA overnight and then diluted to 10 μM and reacted with 300 μM H_2S as above. There was a progressive decrease in PA inhibition of H_2S consumption as the NQ substitutions increased, while PA did not affect H_2S consumption by DMNQ. Values are expressed as the percent of H_2S removed from the solution by NQs. (**G, H**) Effects of overnight incubation of 1 mM 1,4-NQ with 1 mM PA on oxygen consumption (after dilution) by 30 μM 1,4-NQ and 100 μM H_2S (**G**) or 300 μM GSH then 100 μM H_2S (**H**). Oxygen consumption is presented as typical traces.

Figure S6. Effects of H_2S-DCNQ or H_2S-DMNQ adducts on polysulfide production (SSP4 fluorescence). Here, 1 mM NQs were incubated with 1 mM H_2S for 30 min in a closed container that was then opened for 1 h to allow volatilization of the unreacted H_2S and reoxidation of the NQ-SH adduct. The samples were then diluted to either 10 μM or 30 μM NQ and reacted with 10 μM, 100 μM, or 300 μM H_2S in the presence of SSP4, and fluorescence was measured for 100 min. Polysulfide production after the addition of 10 μM H_2S was significantly greater in H_2S pretreated samples of both NQs and for 2,3-DMNQ after the addition of 100 μM or 300 μM 2,3-DMNQ. The effects of 100 μM and 300 μM H_2S on polysulfide production by 2,3-DCNQ were variable. Bar graphs summarize the effects after 100 min of reaction, mean +SE, n=4 wells; *, *p<0.05*, **, *p<0.01*, ***, *p<0.001* vs. NQs without preincubation with H_2S.

Figure S7. Typical traces showing the effects of 300 μM trolox (**A-D**) or tempol (**E-H**) on oxygen consumption in reactions between 300 μM H_2S and 10 μM 1,4-NQ, plumbagin, DMNQ, or DCNQ. Trolox did not affect consumption by any NQ, whereas tempol increased consumption by all NQs except for DMNQ.

Figure S8. Effects of superoxide dismutase (SOD. 0.03 μM) on the percent of H_2S consumed by (**A**) 0.3 μM 1,4-NQ, (**B**) 0.3 μM juglone, (**C**) 3 μM plumbagin, (**D**) 10 μM menadione, (**E**) 1 μM 2-MNQ, (**F**) 1 μM DMNQ, and (**G**) 0.3 μM DCNQ. NQs were incubated with 300 μM H_2S in taped well plates. AzMC (25 μM) was added after 15, 30, 60, 120, or 240 min, and the wells were re-taped and counted at 10 min. Values are expressed as the percent of H_2S removed from the solution; green circles indicate H_2S oxidation by NQ with SOD corrected for H_2S consumption by SOD (blue triangles minus black **X**). (**H, I**) The fold SOD effect, calculated from ((NQ+SOD)/NQ), shows the increase in H_2S consumption by NQs due to SOD at 60 min (**H**) and 120 min (**I**).

Figure S9. LCMS analysis of sulfenylated polysulfides, HS_4OH and HS_5OH, produced by incubation of 100 μM Na_2S, Na_2S_2, Na_2S_3, or Na_2S_4 without or with 10 μM 1,4-NQ, DCNQ, or DMNQ for 20 min at 37 °C in either 21% O_2 (red) or <1% O_2 (blue). (**A**) Comparison as a function of sulfur sources, (**B**) comparison as a function of NQs. (**A**) Mean area under the curve (AUC) +SE, n=3 replicates; *, *p<0.05*, **, *p<0.01*, ***, *p<0.001* vs. respective control (no NQ); x, *p<0.05*, xx, *p<0.01*, xxx, *p<0.001* 1,4-NQ vs. DCNQ or DMNQ; †, *p<0.05*, ††, *p<0.01*, †††, *p<0.001* DCNQ vs. DMNQ; #, *p<0.05*, ##, *p<0.01*, ###, *p<0.001* <1% O_2 vs. 21% O_2.
