# Supplementary material for: Reaction Mechanisms of H2S Oxidation by Naphthoquinones
Source: Antioxidants (Basel). 2024 May 20;13(5):619. doi: 10.3390/antiox13050619 (PMC11117753; doi:10.3390/antiox13050619)
Supplement: Supplementary file 1 [file antioxidants-13-00619-s001.zip › Olson et al supplememtal Figs.pptx]

## Slide 1
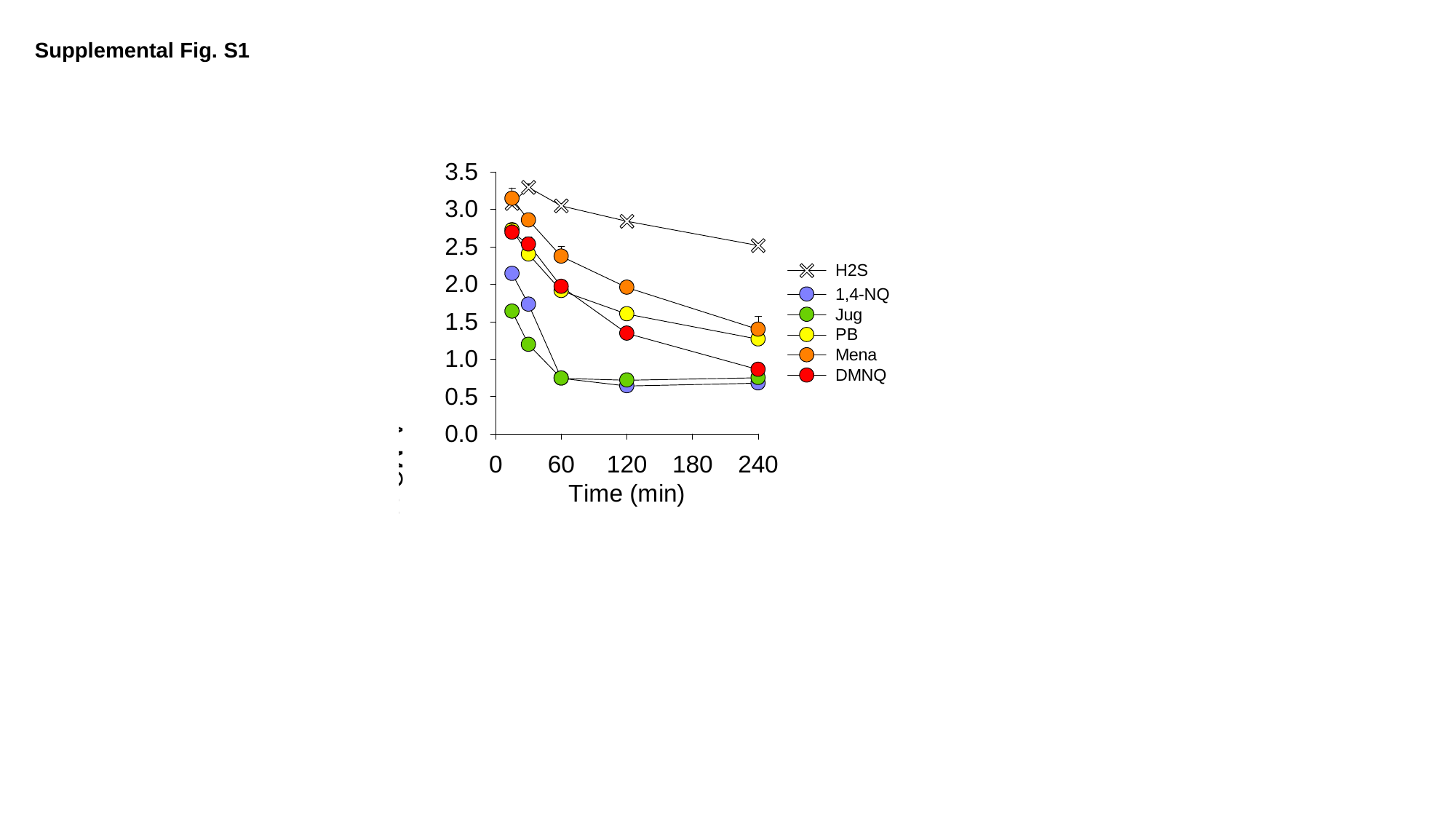

Supplemental Fig. S1

## Slide 2
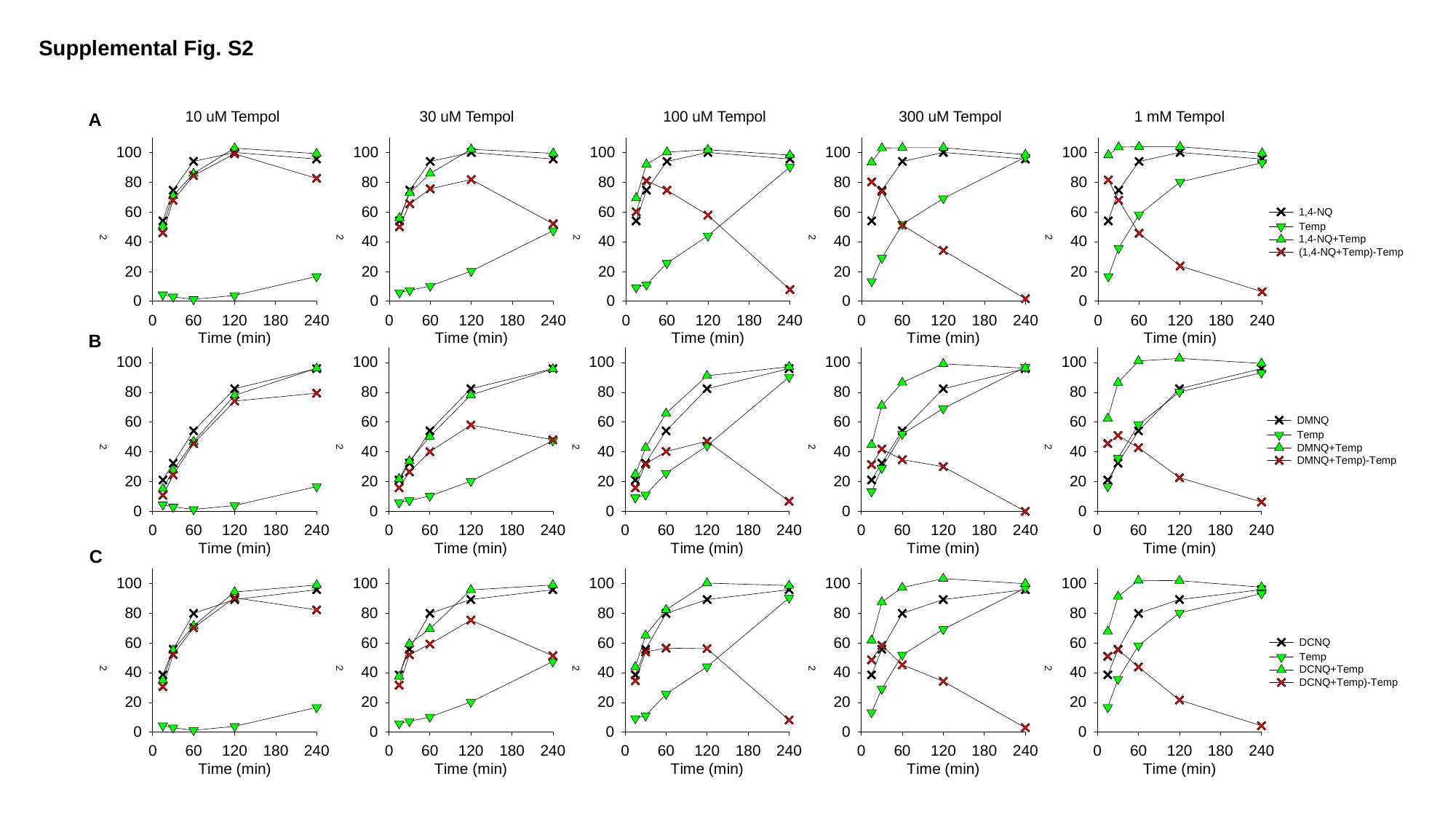

Supplemental Fig. S2
10 uM Tempol
30 uM Tempol
100 uM Tempol
300 uM Tempol
1 mM Tempol
A
B
C

## Slide 3
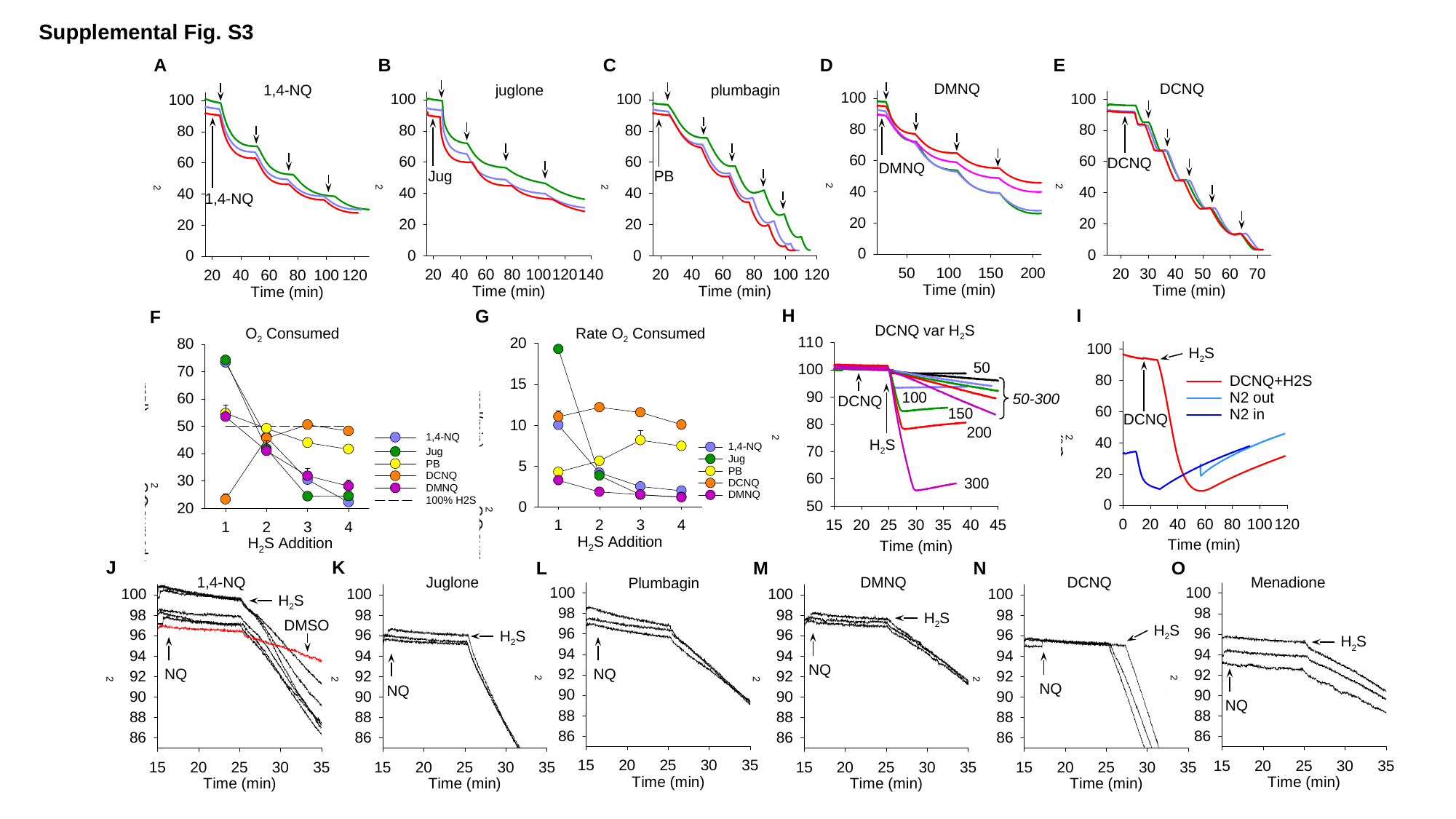

Supplemental Fig. S3
DMNQ
DMNQ
DCNQ
DCNQ
Jug
PB
1,4-NQ
A
B
C
D
E
1,4-NQ
juglone
plumbagin
G
Rate O2 Consumed
F
O2 Consumed
H
50
100
50-300
DCNQ
150
200
H2S
300
DCNQ var H2S
I
H2S
DCNQ
L
Plumbagin
O
Menadione
1,4-NQ
DMSO
J
Juglone
K
DMNQ
M
DCNQ
N
H2S
H2S
H2S
H2S
H2S
NQ
NQ
NQ
NQ
NQ
NQ

## Slide 4
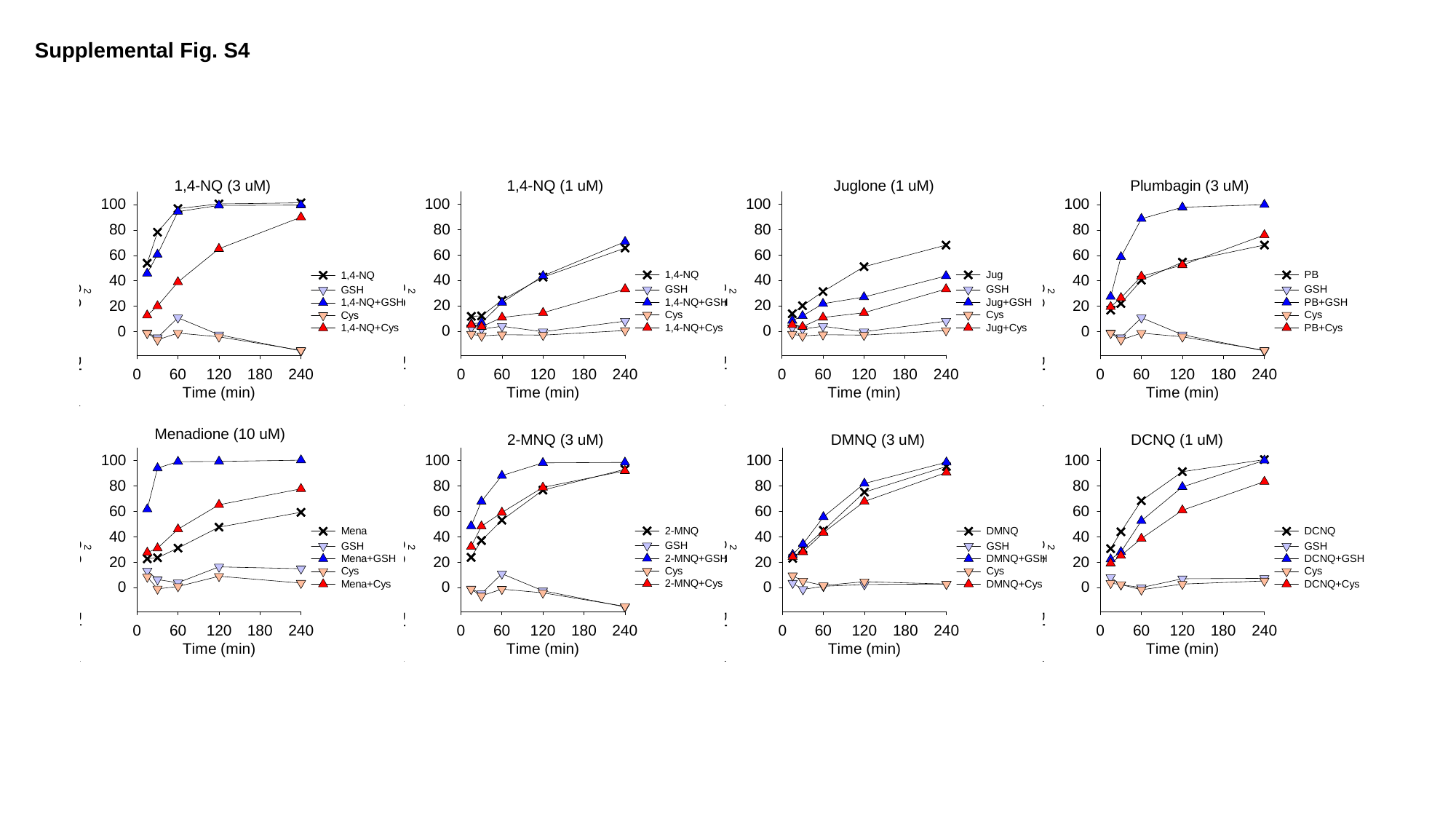

Supplemental Fig. S4
Plumbagin (3 uM)
1,4-NQ (3 uM)
Juglone (1 uM)
1,4-NQ (1 uM)
Menadione (10 uM)
2-MNQ (3 uM)
DMNQ (3 uM)
DCNQ (1 uM)

## Slide 5
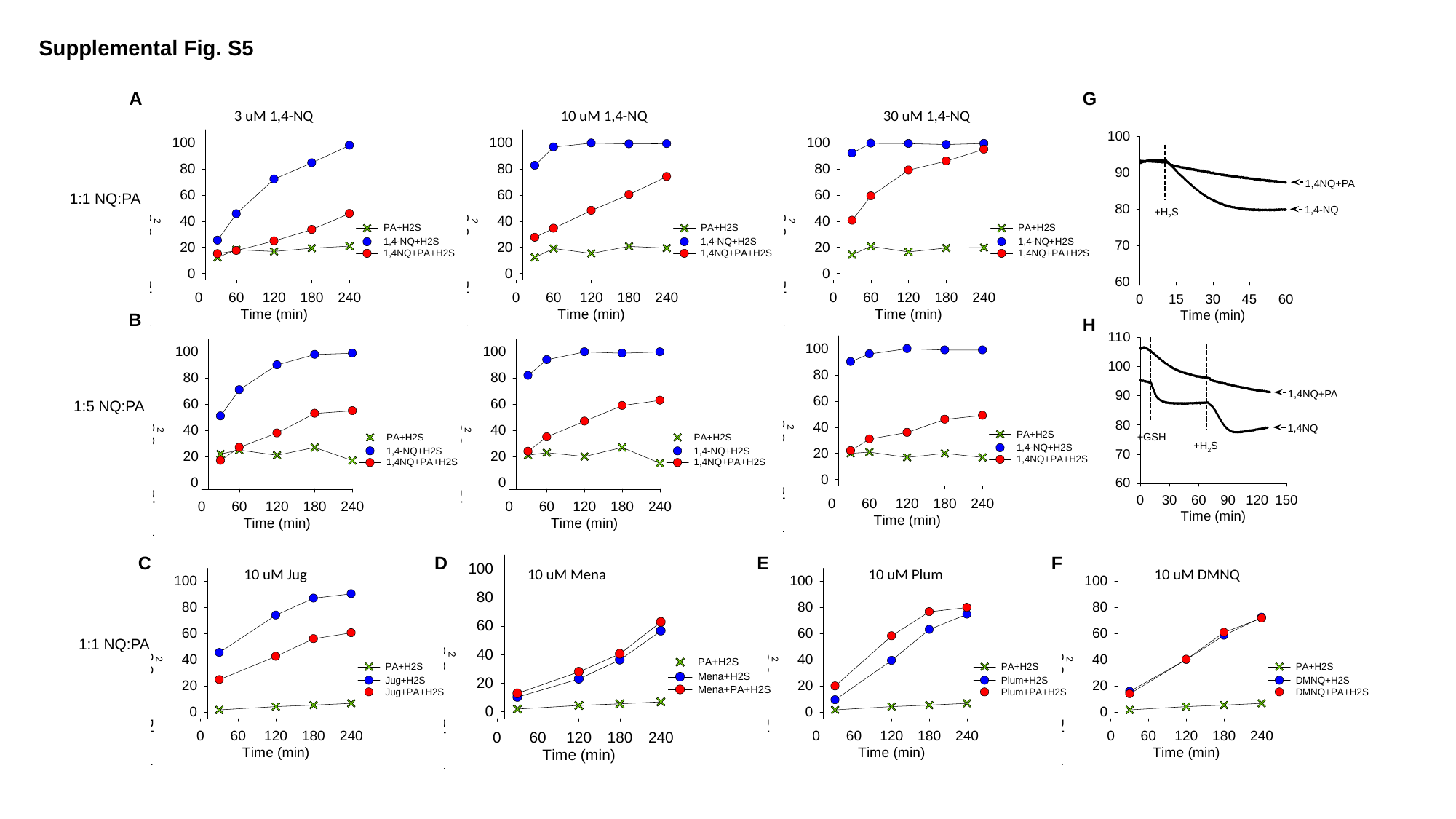

Supplemental Fig. S5
A
G
1,4NQ+PA
1,4-NQ
+H2S
3 uM 1,4-NQ
10 uM 1,4-NQ
30 uM 1,4-NQ
1:1 NQ:PA
1,4NQ+PA
1,4NQ
+GSH
+H2S
B
H
1:5 NQ:PA
C
D
E
F
10 uM Jug
10 uM Mena
10 uM Plum
10 uM DMNQ
1:1 NQ:PA

## Slide 6
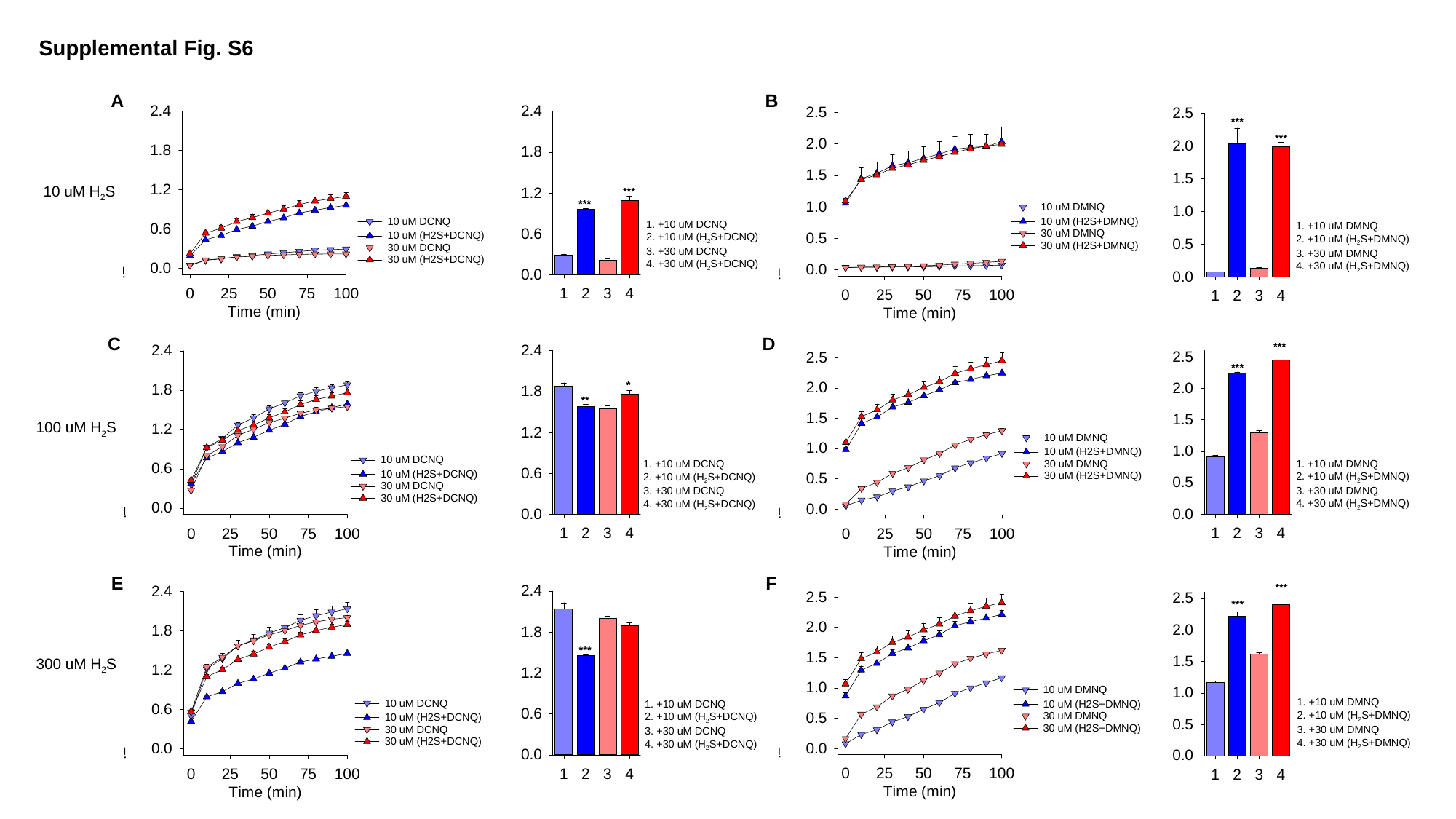

Supplemental Fig. S6
***
***
1. +10 uM DMNQ
2. +10 uM (H2S+DMNQ)
3. +30 uM DMNQ
4. +30 uM (H2S+DMNQ)
***
***
1. +10 uM DMNQ
2. +10 uM (H2S+DMNQ)
3. +30 uM DMNQ
4. +30 uM (H2S+DMNQ)
***
***
1. +10 uM DMNQ
2. +10 uM (H2S+DMNQ)
3. +30 uM DMNQ
4. +30 uM (H2S+DMNQ)
***
***
1. +10 uM DCNQ
2. +10 uM (H2S+DCNQ)
3. +30 uM DCNQ
4. +30 uM (H2S+DCNQ)
*
**
1. +10 uM DCNQ
2. +10 uM (H2S+DCNQ)
3. +30 uM DCNQ
4. +30 uM (H2S+DCNQ)
***
1. +10 uM DCNQ
2. +10 uM (H2S+DCNQ)
3. +30 uM DCNQ
4. +30 uM (H2S+DCNQ)
A
B
10 uM H2S
C
D
100 uM H2S
E
F
300 uM H2S

## Slide 7
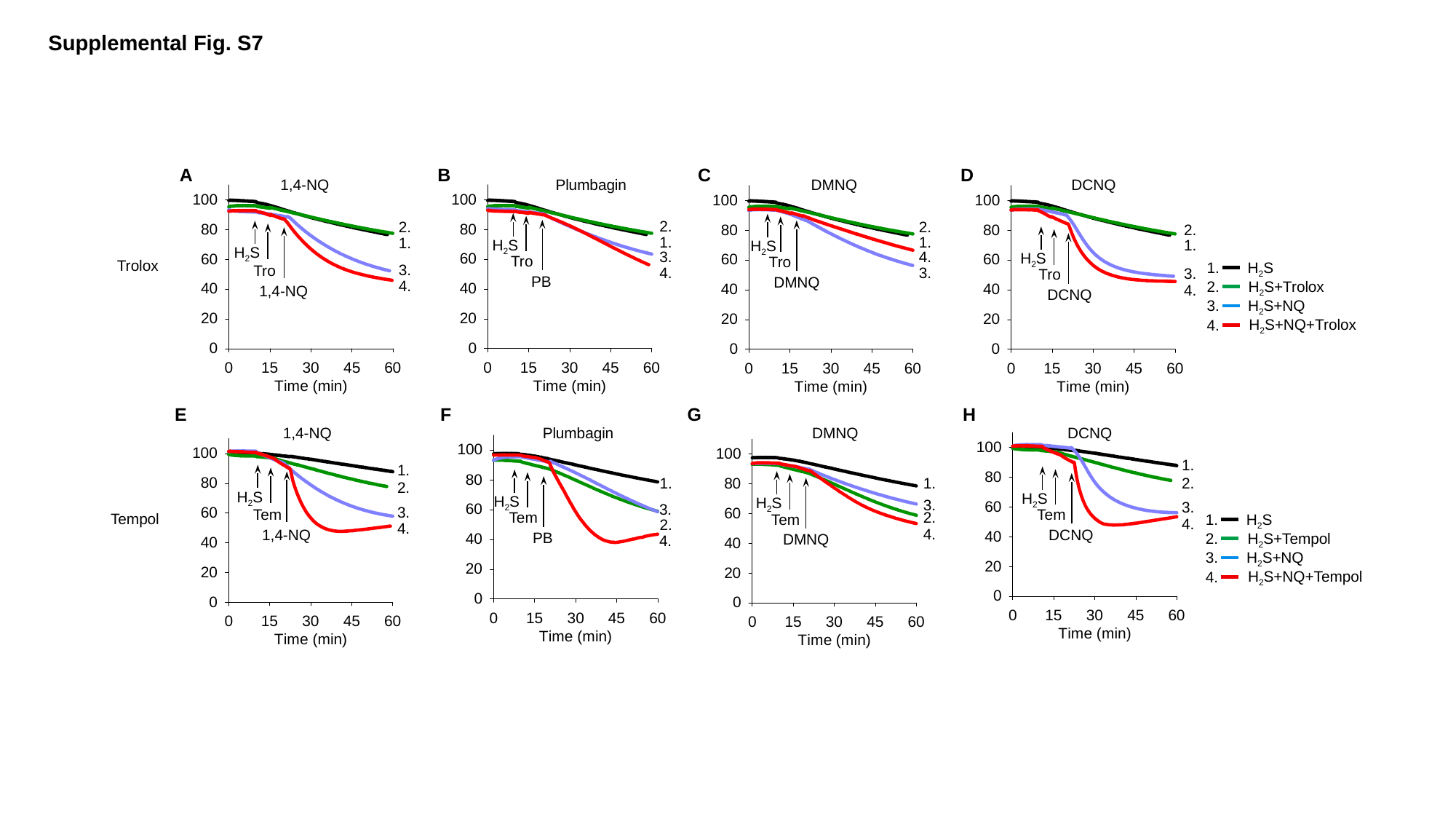

Supplemental Fig. S7
2.
H2S
Tro
PB
1.
3.
4.
2.
H2S
Tro
1,4-NQ
1.
3.
4.
2.
H2S
Tro
DMNQ
1.
4.
3.
2.
H2S
Tro
DCNQ
1.
3.
4.
A
B
C
D
DCNQ
1,4-NQ
Plumbagin
DMNQ
Trolox
1.
2.
3.
4.
H2S
H2S+Trolox
H2S+NQ
H2S+NQ+Trolox
1.
H2S
Tem
DCNQ
2.
3.
4.
1.
H2S
Tem
1,4-NQ
2.
3.
4.
1.
3.
2.
4.
H2S
Tem
DMNQ
E
F
G
H
DCNQ
1,4-NQ
Plumbagin
DMNQ
1.
H2S
Tem
PB
3.
2.
4.
Tempol
1.
2.
3.
4.
H2S
H2S+Tempol
H2S+NQ
H2S+NQ+Tempol

## Slide 8
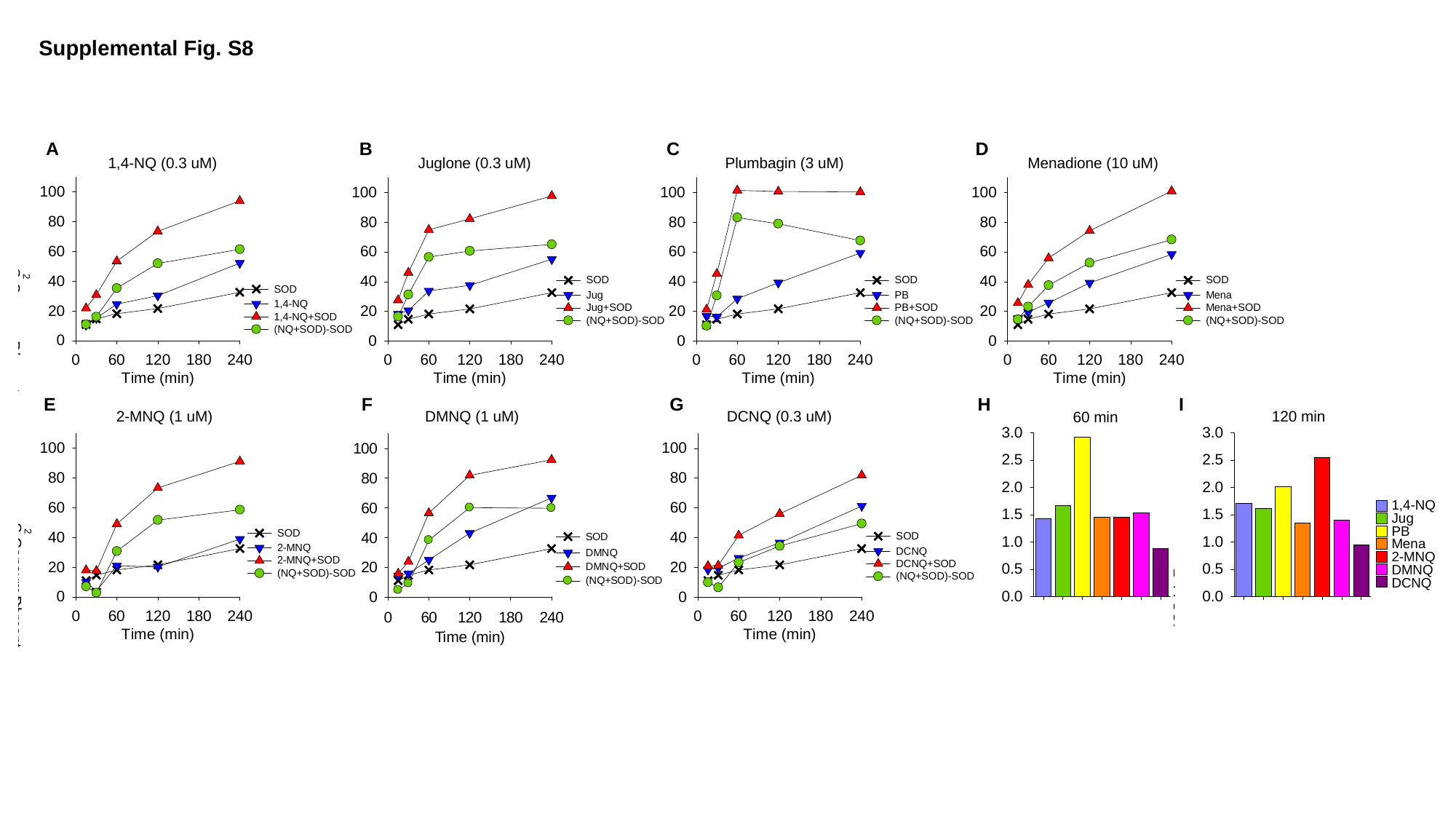

Supplemental Fig. S8
A
B
C
D
1,4-NQ (0.3 uM)
Juglone (0.3 uM)
Plumbagin (3 uM)
Menadione (10 uM)
E
F
G
H
I
2-MNQ (1 uM)
DMNQ (1 uM)
DCNQ (0.3 uM)
120 min
60 min
1,4-NQ
Jug
PB
Mena
2-MNQ
DMNQ
DCNQ

## Slide 9
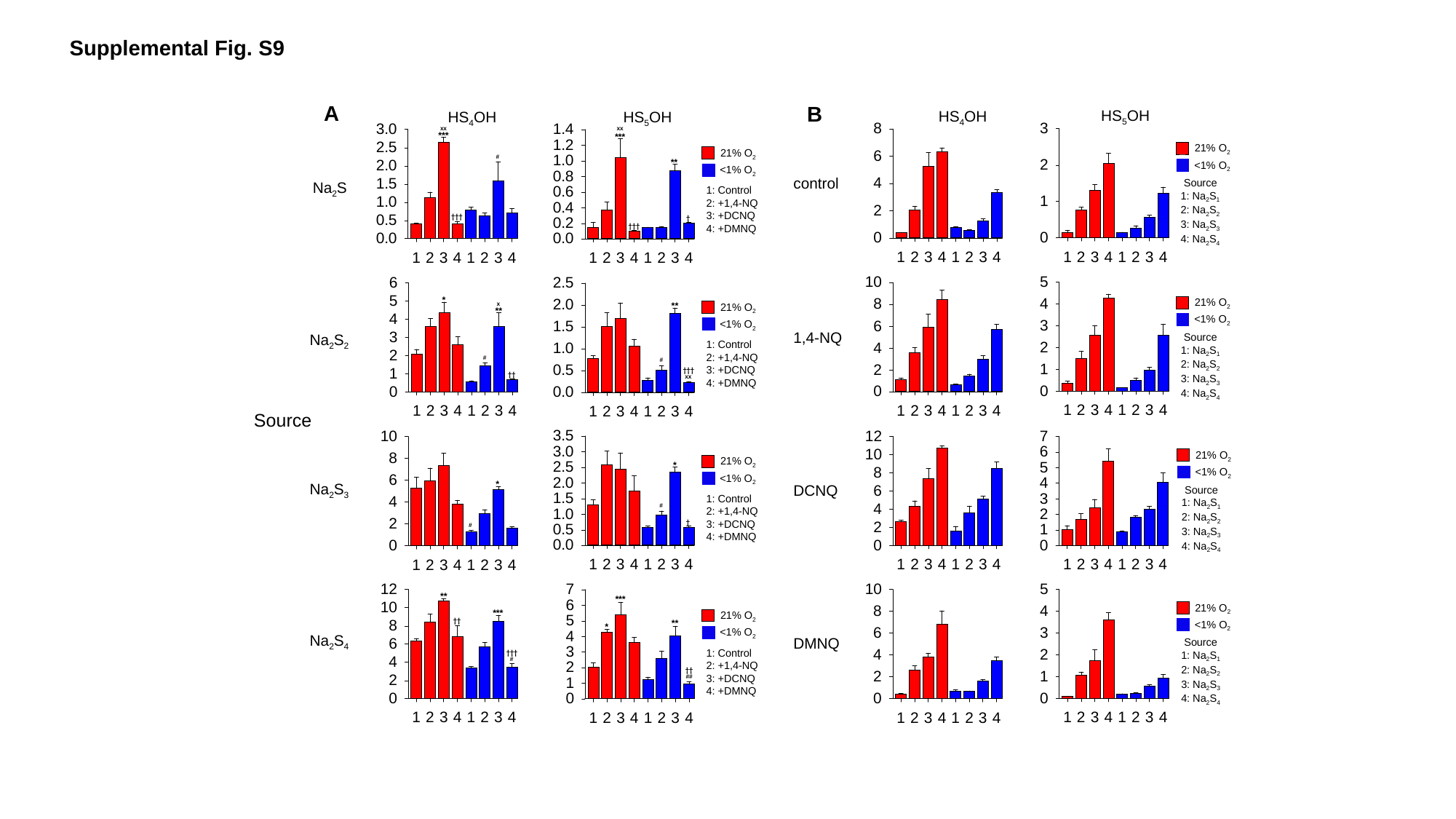

Supplemental Fig. S9
xx
***
#
†††
xx
***
**
†
†††
A
B
HS5OH
HS4OH
HS4OH
HS5OH
21% O2
<1% O2
 Source
1: Na2S1
2: Na2S2
3: Na2S3
4: Na2S4
21% O2
<1% O2
1: Control
2: +1,4-NQ
3: +DCNQ
4: +DMNQ
21% O2
<1% O2
1: Control
2: +1,4-NQ
3: +DCNQ
4: +DMNQ
21% O2
<1% O2
1: Control
2: +1,4-NQ
3: +DCNQ
4: +DMNQ
21% O2
<1% O2
1: Control
2: +1,4-NQ
3: +DCNQ
4: +DMNQ
control
1,4-NQ
DCNQ
DMNQ
Na2S
*
x
**
#
††
**
#
†††
xx
21% O2
<1% O2
 Source
1: Na2S1
2: Na2S2
3: Na2S3
4: Na2S4
Na2S2
*
#
†
*
#
Source
21% O2
<1% O2
 Source
1: Na2S1
2: Na2S2
3: Na2S3
4: Na2S4
Na2S3
**
***
††
†††
#
***
**
*
††
##
21% O2
<1% O2
 Source
1: Na2S1
2: Na2S2
3: Na2S3
4: Na2S4
Na2S4
